# Betaine Attenuates Hyperhomocysteinemia-Induced Cognitive Impairment by Suppressing Oxidative Stress and Activating the PI3K/AKT/GSK-3β Pathway

**DOI:** 10.3390/antiox15070807

**Published:** 2026-06-27

**Authors:** Xiaolong Gu, Yuan Fu, Yongli Zhao, Zhenyi Liu, Yixiao Yang, Qi Xie, Peng Ma, Zhiwei Peng, Zhizhen Liu, Jianting Li, Jun Xie

**Affiliations:** 1 Shanxi Key Laboratory of Birth Defect and Cell Regeneration, Department of Biochemistry and Molecular Biology, Shanxi Medical University, Taiyuan 030001, China; gxl04022022@126.com (X.G.); fy1009706220@163.com (Y.F.); 18834112074@163.com (Y.Z.); 19544545890@163.com (Y.Y.); 15735258351@163.com (Q.X.); mapeng@sxmu.edu.cn (P.M.); zhwp2022@sxmu.edu.cn (Z.P.); zhizhenliu2013@163.com (Z.L.); 2 Key Laboratory of Coal Environmental Pathogenicity and Prevention, Ministry of Education, Shanxi Medical University, Taiyuan 030001, China; 3 School of Pharmaceutical Science, Shanxi Medical University, Taiyuan 030001, China; liuzhenyi1228@163.com

**Keywords:** betaine, homocysteine, ROS, cognitive impairment, PI3K/AKT/GSK-3β pathway

## Abstract

High homocysteine levels are a key risk factor for cognitive impairment, a major public health concern in aging societies. Although betaine is known to reduce Hcy levels, its effects on hyperhomocysteinemia (hHcy)-induced cognitive impairment and the underlying mechanisms remain unclear. Here, we established an hHcy-induced cognitive impairment mouse model by feeding mice a high-methionine diet for 8 weeks, followed by betaine supplementation for 14 days. Betaine treatment attenuated hHcy-induced cognitive impairment. This improvement was accompanied by alleviation of neuropathological alterations and enhancement of antioxidant capacity. Notably, betaine suppressed reactive oxygen species (ROS) accumulation, neuronal apoptosis, and Tau hyperphosphorylation at Ser396 and Thr231 in both mouse hippocampus and HT-22 cells. Mechanistically, betaine-induced activation of the PI3K/AKT/GSK-3β pathway was effectively blocked by the PI3K inhibitor LY294002. Notably, treatment with the ROS scavenger N-acetylcysteine (NAC) alone phenocopied this activation, suggesting that ROS functions as an upstream regulator of this signaling cascade. Collectively, our data demonstrate that betaine attenuates hHcy-induced cognitive impairment by suppressing oxidative stress-driven apoptosis and Tau pathology through modulation of the PI3K/AKT/GSK-3β signaling pathway. These findings suggest that betaine may hold promise for further preclinical and clinical studies, although long-term efficacy and safety evaluations remain necessary.

## 1. Introduction

Dementia encompasses a group of progressive brain disorders characterized by deterioration in cognitive and behavioral functions, including memory loss, compromised communication and language abilities, diminished attention and concentration, deficits in reasoning and judgment, and visual perceptual abnormalities [1]. Alzheimer’s disease (AD), the most common form of dementia, accounts for approximately 60–80% of all cases [2]. Precisely preventing and controlling important risk factors may help prevent or slow the progression of dementia [3]. Several potential risk factors for cognitive decline or dementia, such as age, genetics, hypertension, and lipid metabolism disorders, have been identified [4,5,6]. Epidemiological studies further identify elevated plasma total homocysteine as a modifiable risk factor for AD [7,8,9]. Although drugs have been developed to address cognitive impairments, their efficacy and safety require evaluation. Currently, no disease alleviation or prevention strategies are available in this context.

In recent years, the metabolism of homocysteine (Hcy) has gradually been recognized as a risk factor for many cardiovascular diseases. As a key intermediate in methionine and cysteine metabolism, Hcy can be synthesized physiologically in all cells [10]. Congenital metabolic abnormalities can lead to hHcy, and hcy accumulation damages the vascular system and promotes neurodegenerative changes, ultimately resulting in cognitive decline [10,11,12]. Although hHcy is closely related to various neurological diseases, the molecular mechanism of its neurotoxicity has not yet been fully elucidated. In the general population, plasma Hcy concentrations typically range from 5 to 15 μmol/L, with levels above 15 μmol/L defining as hyperhomocysteinemia [13,14]. A meta-analysis found that each 5 μmol/L rise in plasma Hcy was associated with a 9% higher risk of dementia and a 12% risk of AD [15]. Therefore, monitoring cumulative Hcy exposure is of key importance for assessing the risk of dementia and formulating targeted intervention strategies.

The etiology of dementia involves a complex interplay of mechanisms. Aberrant neuroinflammation, autophagy, and apoptosis are recognized as critical drivers of neuronal cell damage, leading to cerebrovascular disease and cognitive dysfunction [16]. hHcy have been identified as significant risk factors for dementia through diverse pathways, such as oxidative stress [17,18], cerebrovascular damage [19], endoplasmic reticulum (ER) stress [20], Aβ pathology [21,22], and Tau protein hyperphosphorylation [23]. The PI3K/AKT signaling pathway is a fundamental regulator of cellular homeostasis and controls processes such as cell proliferation, survival, and apoptosis [24]. Its dysregulation is a key contributor to cellular dysfunction. A major downstream target of AKT is glycogen synthase kinase-3β (GSK3β). AKT-mediated phosphorylation inhibits GSK3β activity, thereby blocking its pro-apoptotic functions [25]. Importantly, this inactivation of GSK3β plays a crucial role in mitigating core AD pathologies by reducing Aβ production and preventing the abnormal hyperphosphorylation of Tau protein [26,27].

Betaine (also referred to as N,N,N-trimethylglycine) is a naturally existing quaternary ammonium compound present in dietary sources including beets, spinach, and whole grains [28]. It is transported primarily to the central nervous system through betaine-GABA transporter protein (BGT-1) [29,30]. The core physiological function of betaine is to act as a methyl donor in the methylation reaction, promoting the conversion of Hcy to methionine, thereby effectively decreasing plasma Hcy levels and reducing the risk of diseases associated with hHcy [31]. Some evidence supports the effectiveness of supplementation with betaine in the context of reducing the concentration of Hcy in the blood circulation [32,33,34,35]. Recent studies have identified multiple pathways through which betaine exerts neuroprotective effects, including direct inhibition of TBK1, suppression of NLRP3-mediated pyroptosis via m6A-YTHDF2 [36,37]. However, whether betaine ameliorates high-methionine diet-induced cognitive impairment specifically through the ROS/PI3K/AKT/GSK-3β/tau axis has not been systematically investigated.

In this study, we observed that mice fed an HMD exhibited elevated Hcy levels and subsequent cognitive impairment. Supplementation with betaine in the drinking water restored cognitive function. To further explore the molecular mechanism underlying this improvement, we conducted mechanistic studies in both animals and cells. These findings provide novel evidence that supports the clinical use of betaine and the design of relevant drug targets or small molecules compounds for treating cognitive impairment.

## 2. Materials and Methods

### 2.1. Cell Culture and Hcy Treatment

The mouse hippocampal neuron cell line (HT-22; purchased from Jennio bioicol Technology in Guangzhou, China) was grown in Dulbecco’s modified Eagle’s medium (DMEM) containing 10% fetal bovine serum, 1% streptomycin-penicillin solution, and 1% nonessential amino acids. The cells were divided into the CON group (cultured normally for 24 h), the HTL group (treated with 2 mM HTL for 24 h), and the betaine group (pretreated with 10 mM betaine for 4 h and then treated with 2 mM homocysteine thioacetamide (HTL) for 24 h) [37].

### 2.2. Cell Counting Kit-8

Cell proliferation was measured using a Cell Counting Kit-8 (BA00208, Bioss, Beijing, China). Cells were seeded in 96-well plates (H803004, BDBIO, Shanghai, China; 5 × 10^3^ cells per well), and after they reached 70% confluence, they were treated for 24 h. 10 μL CCK-8 solution and 90 μL culture medium were added per well. After incubation for 1 h, the absorbance was measured at 450 nm using an enzyme-linked spectrophotometer (Molecular Devices, Sunnyvale, CA, USA).

### 2.3. Crystal Violet Staining

The HT-22 cells were fixed with 4% paraformaldehyde for 10 min, then rinsed with phosphate-buffered saline (PBS; Servicebio, Wuhan, China), and then incubated with crystal violet staining solution (C0121; Beyotime, Shanghai, China) for 10 min. Images were acquired with an inverted microscope (Nikon, Tokyo, Japan).

### 2.4. Apoptosis Detection

The apoptosis rate of the cells was detected using an Annexin V-EGFP/PI apoptosis detection kit (KTA0005, Abbkine, Wuhan, China). After each group of HT-22 cells was treated for 24 h, they were digested with trypsin without EDTA. After being washed with PBS, the cells were resuspended in 100 μL of Annexin V binding buffer. Annexin V-FITC and PI staining was performed, and flow cytometry (BD FACSCelesta, Franklin Lake, NJ, USA) was used for detection. Apoptosis rate was calculated as the total of early apoptotic cells located in lower-right quadrant and late apoptotic cells located in upper-right quadrant.

### 2.5. Measurement of ROS Content in Cells

The level of reactive oxygen species (ROS) was determined using 2,7-dichlorodihydrofluorescein (DCFH-DA), which is a specific ROS-specific fluorescent probe. After the cells were treated with betaine and HTL, the DCFH-DA probe was diluted to 10 µM. The cells were harvested and resuspended in diluted DCFH-DA solution at a density of 1 to 2 million per milliliter. The cells were incubated in a 37 °C cell culture box for 20 min. Every 3–5 min, the mixture was inverted and mixed well to ensure that the probe and the cells were in full contact. We removed unincorporated DCFH-DA by washing the cells three times with serum-free DMEM. Then, we analyzed cells by flow cytometry at 488 nm (excitation) and 525 nm (emission).

### 2.6. RNA-Seq

Total RNA was isolated from three groups of HT-22 cells using the standard TRIzol protocol. mRNA was captured using oligo (dT) magnetic beads, and library were constructed for RNA-seq. High-throughput sequencing was conducted on an Illumina HiSeq X Ten or NovaSeq 6000 platform (Illumina, San Diego, CA, USA).

Clean reads were mapped to the reference genome with HISAT2. FPKM values and read counts were determined using HTSeq-count. Principal component analysis (PCA; R v3.2.0) was performed to evaluate consistency among biological replicates.

Differential expression analysis was conducted using DESeq2 [38]. Differentially expressed genes (DEGs) were considered as those with a Q value < 0.05 and a fold change > 2 or <0.5. Hierarchical clustering of DEGs (R; v3.2.0) was used to visualize expression patterns. The top up- and downregulated DEGs were visualized as a radar map created with the R package ggradar.

Based on the hypergeometric distribution, GO [39], KEGG [40] pathway enrichment analyses of the DEGs were conducted to identify significantly enriched terms with R (v 3.2.0). Column and bubble charts of the significant terms were generated with R.

### 2.7. Animals

To avoid variability from estrous-cycle hormones and establish a clear mechanism, we used male mice, which offer a more homogeneous model for assessing betaine’s neuroprotection. Specific pathogen-free 18–20 g C57BL/6 adult male mice (8 weeks old) were purchased from the Animal Center of Shanxi Medical University. The mice were divided into three groups, with six animals in each group: (1) the Con group was fed a standard diet (main components consisting of 23.07% protein, 11.85% fat, and 65.08% carbohydrates; Beijing Keaoxieli Feed Co., Ltd., Beijing, China); (2) the HMD group was fed a high-methionine diet (main components including 2% methionine; Beijing Keaoxieli Feed Co., Ltd.) for 8 weeks; and (3) the Bet + HMD group was fed a high-methionine diet (main components consisting of 2% methionine; Beijing Keaoxieli Feed Co., Ltd.) [41] for 8 weeks, after which betaine (2.5% *w*/*v*) was supplemented in the drinking water (for 2 weeks). Water bottles were replaced weekly, with each cage (2 mice) receiving 100 mL of fresh solution. Water consumption was measured by weighing the bottles, yielding an average intake of 4.7–5.5 mL/mouse/day, which corresponds to a daily betaine dose of 4.1–5.4 g/kg body weight. All animal experiments were conducted in the barrier environment of the Animal Experiment Center of Shanxi Medical University. Each cage contained 2–3 animals, with a constant temperature of 24 ± 2 °C, a 12 h/12 h light-dark cycle, and a constant humidity of 40–70%.

### 2.8. Morris Water Maze Test

Long-term spatial learning and memory were evaluated in mice using the Morris water maze test. The experiment was conducted in a 120 cm-diameter white circular water tank, which was filled with water at a temperature of 22 ± 2 °C and a depth of approximately 25 cm. We placed a circular platform in the northwest quadrant with 1 cm below the water surface, and a video system automatically recorded data for each mouse.

The experiment consisted of four days of training and a test on the fifth day. During the training period, the mice were placed in water in different quadrants, facing the wall, and subjected to a 60 s test. If a mouse found the platform within 60 s and remained on it for 3 s, the trial ended and escape latency (time to find the platform) was recorded. Mice that failed to locate the platform were directed onto it and left there for 20 s, their latency was recorded as 60 s. The daily performance was averaged across the four quadrants. On the fifth day, the platform was removed, and the latency for the mouse to cross the platform for the first time and the number of times it crossed the platform within 60 s were measured.

### 2.9. T-Maze Test

Spontaneous activity and voluntary choices were accessed using a T-maze to evaluate the short-term spatial learning and memory. T-maze included three arms (40 × 8 × 15 cm). At the beginning of the experiment, mice were positioned in the starting box at the end of the start arm.

New arm exploration test: During training, the novel arm was blocked, and the mice were obstructed, and the mice were permitted to roam the maze unrestrictedly for 10 min. One hour later, the barrier was removed, and the mice explored for 5 min. The number of entries into the new arm was recorded.

### 2.10. Protein Extraction and Western Blotting

For HT-22 cells, total protein was extracted with RIPA lysis buffer (PC101, EpiZyme, Shanghai, China). The protein concentration was detected by the BCA method. Protein samples were separated by 8–15% SDS-PAGE, transferred to PVDF membrane (IPVH00010, Millipore, Boston, MA, USA). The membrane was blocked with 5% BSA at room temperature for 2 h and then incubated with the primary antibody at 4 °C overnight. The next day, the membrane was washed and incubated with goat anti-rabbit IgG (H + L) (1:10,000, bs-80295G-HRP, Bioss, Beijing, China) at room temperature for 1 h. The immune reaction bands were detected using an enhanced chemiluminescence (ECL) substrate, the images were collected using a ChemiDoc XRS system (Bio-Rad, Hercules, CA, USA), and the immunoblotting density was quantified using ImageJ software (version 1.8.0.112; National Institutes of Health, Bethesda, MD, USA). Relative protein expression was normalized to that of GAPDH (total protein) or β-actin (total protein). The antibodies used were as follows: rabbit anti-Tau-5 (1:1000; Beyotime, Shanghai, China, Cat# AF1249), rabbit anti-pS199-Tau (1:1000; Beyotime, Cat# AG2590), rabbit anti-pT231-Tau (1:1000; Beyotime, Cat# AF1951), rabbit anti-pS396-Tau (1:1000; Beyotime, Cat# AF2368), rabbit anti-GSK3β (1:1000; CST, Boston, MA, USA, Cat# D5C5Z), rabbit anti-pS9-GSK3β (1:1000; Wanleibio, Shenyang, China, Cat# WL03683), mouse anti-PI3 kinase p85 alpha (1:1000; Zenbio, Chengdu, China, Cat# 251221), rabbit anti-phospho-PI3 kinase p85/p55 (1:1000; Zenbio, Cat# 251221), rabbit anti-phospho-AKT (Ser473) (1:1000; Zenbio, Cat# 310021), rabbit anti-AKT (1:1000; Proteintech, Wuhan, China, Cat# 10176-2-AP), rabbit anti-caspase3 (1:1000; Proteintech, Cat# 19677-1-AP), rabbit anti-Bax (1:20,000, Proteintech, Cat# 50599-2-lg), rabbit anti-Bcl-2 (1:5000, HUABIO, Hangzhou, China, Cat# ET1702-53), rabbit anti-beta actin(1:5000, Proteintech, Cat# 20536-1-AP), mouse-anti-β-Tubulin Mouse Monoclonal Antibody (1:5000, TransGen Biotech, Beijing, China, Cat# HC101-01), rabbit anti-AT8(Ser202/Thr205) (1:10,000, Proteintech, Cat# 82568-1-RR), rabbit anti-AT100(Thr212/Ser214) (1:2000, Proteintech, Cat# 87103-1-RR) and rabbit anti-GAPDH (1:10,000, Proteintech, Cat# 10494-1-AP).

### 2.11. Metabolomics Analysis

Metabolomics was performed ultra-performance liquid chromatography coupled with high-resolution mass spectrometry (LC-MS/MS) technology to maximize the amount of total metabolite information obtained from the mouse hippocampus samples.

UHPLC-MS/MS was conducted on a Vanquish UHPLC system (Thermo Fisher, Waltham, MA, USA) linked to an Orbitrap Q Exactive HF or HF-X mass spectrometer (Thermo Fisher, Germany) at Novogene Co., Ltd. (Beijing, China). Separation was performed on a Hypersil Gold column (100 × 2.1 mm, 1.9 μm) at 0.2 mL/min with a 12 min linear gradient. Mobile phases consisted of 0.1% FA in water (A) and methanol (B). The gradient profile was: 2% B at 0–1.5 min, 2–85% B from 1.5 to 4.5 min, 85–100% B from 4.5 to 10 min, then back to 2% B at 10.1 min and held until 12 min. The Q Exactive HF mass spectrometer was operated in both positive and negative ion modes. Key settings included a spray voltage of 3.5 kV, capillary temperature of 320 °C, sheath gas flow of 35 psi, auxiliary gas flow of 10 L/min, S-lens RF level of 60, and auxiliary gas heater temperature of 350 °C.

Using Compound Discoverer 3.3 (Thermo Fisher), we processed the raw UHPLC-MS/MS data to align peaks, select features, and quantify metabolites. The main parameters were first-QC-corrected peak area, 5 ppm mass tolerance, 30% signal intensity tolerance, and a minimum intensity cutoff. Following normalization to total spectral intensity, the data were used to predict molecular formulas based on adduct, molecular, and fragment ions. Peak matching against mzCloud, mzVault, and mass list databases provided qualitative and relative quantitative results. Statistical analyses were conducted using R (3.4.3), Python (2.7.6), and CentOS (6.6). For non-normally distributed data, standardization was performed using the formula: raw quantitation value/(sum of sample quantitation values/sum of QC1 sample quantitation values). This yielded relative peak areas. Compounds whose relative peak area CV > 30% in QC samples were discarded. Finally, identification and the relative quantification of metabolites were obtained.

Metabolite annotation was carried out using the KEGG database (https://www.genome.jp/kegg/pathway.html, accessed on 25 June 2026). PCA and PLS-DA were conducted using metaX (v2.90), a comprehensive metabolomics data processing software. Univariate *t*-tests were used to calculate *p* values. Metabolites with VIP > 1, *p* < 0.05, and fold change ≥ 2 or ≤0.5 were considered differentially abundant. Volcano plots were generated using the ggplot2 package in R, plotting log2(fold change) against −log10(*p* value) to filter candidate metabolites.

### 2.12. Enzyme-Linked Immunosorbent Assay for Homocysteine

The HCY concentration in serum, brain tissue, and cells was determined using a Mouse Hcy enzyme-linked immunosorbent assay (ELISA) Kit (LV30834M; Animalunion Biotechnology, Shanghai, China).

### 2.13. Hematoxylin–Eosin Staining and Nissl Staining

Hematoxylin–eosin (HE) staining (G1003; Servicebio) and Nissl staining (G1036; Servicebio) were used to observe pathological changes in the cortex and hippocampus under an optical microscope (Olympus, Tokyo, Japan).

### 2.14. Statistical Analyses

GraphPad Prism 9 statistical software (San Diego, CA, USA, www.graphpad.com) was used. Normality and variance homogeneity were assessed using the Shapiro–Wilk test and Levene’s test, respectively. Comparisons between two groups were performed using the independent-sample *t* test. For comparisons among three or more groups, one-way analysis of variance (ANOVA) followed by Tukey’s post hoc test was applied for normally distributed data, while the Kruskal–Wallis test with Dunn’s post hoc test was used for non-normally distributed data. Results are expressed as mean ± standard deviation (SD) or median (interquartile range), with *p* < 0.05 considered statistically significant.

## 3. Results

### 3.1. Betaine Alleviates HMD-Induced Cognitive Impairment in Mice

**Figure 1:**
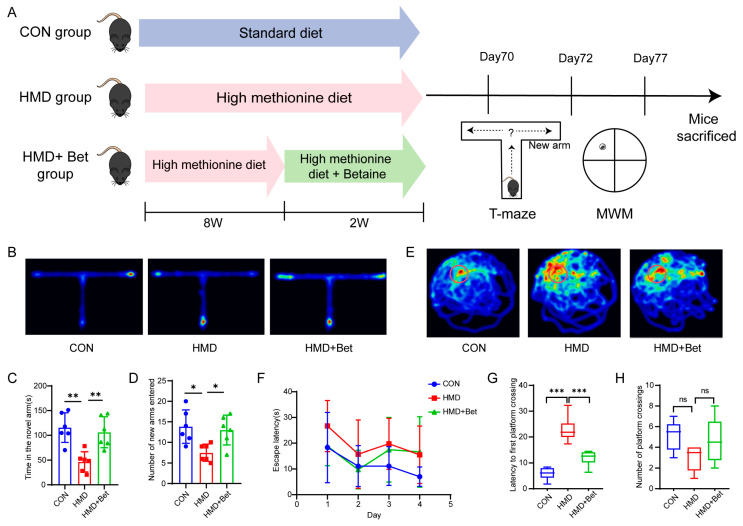
Betaine alleviates high methionine diet-induced cognitive impairment in mice. (**A**) Schematic diagram of the animal experiments. (**B**) Heat maps of the T maze test in control and high methionine diet group with or without betaine treatment. (**C**,**D**) The time and the number of new arms entered by the control or high methionine diet groups with or without betaine rescue mice in the T maze test (*n* = 6). (**E**) Group average heatmap of the swimming trajectories of the control or high methionine diet groups mice with or without betaine rescue mice in Morris water maze tests (*n* = 6). Red circles indicate the location of the platform. (**F**–**H**) Escape latency during the training period, latency to first platform crossing and number of platform location crosses during the Morris water maze experimental period (*n* = 6). CON denotes control group. HMD denotes high-methionine diet mice and HMD + BET denotes high-methionine diet mice with betaine rescue. * *p* < 0.05, ** *p* < 0.01, *** *p* < 0.001; ns, not significant, (one-way ANOVA or Kruskal–Wallis test).

We fed C57BL/6 male mice with an HMD for 2 months to establish an hHcy model [42]. Then, we treated the mice with betaine in their drinking water for 2 weeks to investigate the proactive effect of betaine on cognitive function (Figure 1A). Learning and memory abilities were evaluated using the T-maze and Morris water maze tests. The heatmap revealed the differences in exploration behavior among the groups during the T-maze test (Figure 1B). Compared with the control group, the HMD group showed significant decreases in both the time and the number of entries into the new arm. In contrast, compared with the HMD group, the betaine group showed significantly increased exploration of the new arm, along with an increased time and number of entries into the new arm (Figure 1C,D). Furthermore, during the training phase of the Morris water maze test, the HMD-fed mice learned the platform location at a slower rate. Moreover, during the testing phase, compared with those in the control group, the escape latency in the HMD group was significantly longer, and the number of platform crossings was reduced. However, betaine treatment ameliorated hyperhomocysteinemia-induced effects (Figure 1E–H), whereas betaine treatment alone did not show any significant improvements (Figure S1). In conclusion, these results confirmed that compared with the control mice, HMD-fed mice exhibited long-term memory decreased, which indicated that an increase in the Hcy concentration led to cognitive impairment and that betaine could ameliorate the cognitive impairment induced by hHcy.

### 3.2. Betaine Attenuates High Methionine Diet-Induced Brain Injury in Mice

**Figure 2:**
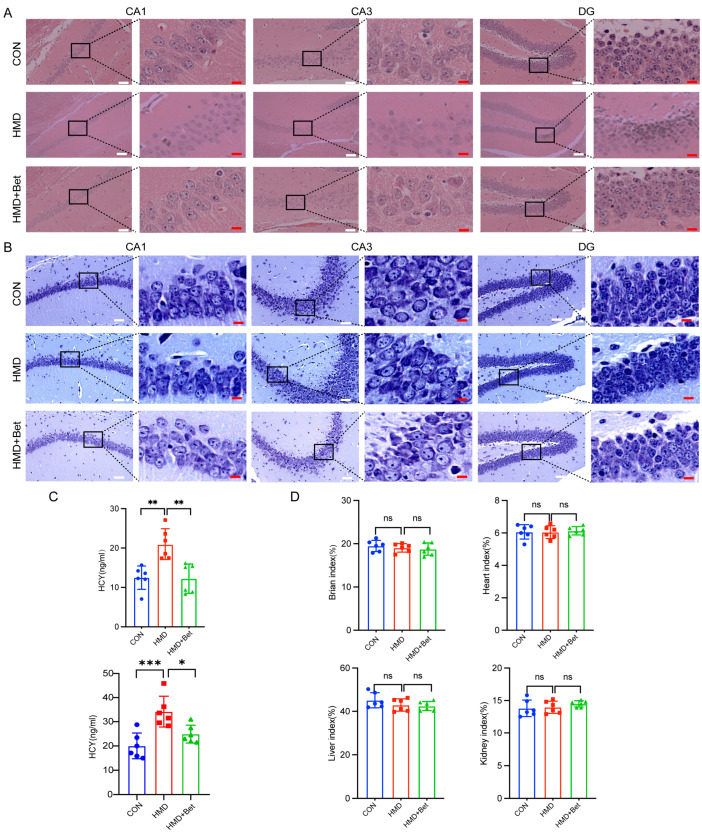
Betaine attenuates high methionine diet-induced brain injury in mice. (**A**,**B**) Representative H&E staining and Nissl staining images of hippocampus CA1, CA3 and DG area in the control or high methionine diet groups mice with or without betaine rescue. Scale bars: red, 25 μm; white, 50 μm. Dashed boxes indicate the regions that are shown at higher magnification in the adjacent panels. (**C**) Hcy levels in the serum (**upper** panel) and brain tissue (**down** panel) of control, high-methionine diet mice with or without betaine rescue were measured via ELISA. (**D**) The organ indices for brain, heart, liver and kidney of control, high-methionine diet mice with or without betaine rescue (*n* = 6). * *p* < 0.05, ** *p* < 0.01, *** *p* < 0.001; ns, not significant, (one-way ANOVA).

To evaluate the protective effect of betaine on cognitive impairment, we assessed histopathological changes in the CA1, CA3, and DG regions of the hippocampus by Nissl and H&E staining. In the H&E-stained sections, the brain neurons of the control group showed intact morphology and a dense arrangement, with no obvious neuronal loss. In contrast, the HMD group exhibited a sparse cell arrangement and pathological changes such as cell body shrinkage and cell membrane rupture. In the betaine treatment group, the number of neurons partially recovered, and the cell arrangement hierarchy and morphology were improved (Figure 2A). The results of Nissl staining also indicated that the neurons in control group in the CA1, CA3, and DG regions exhibited normal morphology, clear structure, and orderly cell arrangement. While in the HMD group, abnormal changes, such as widened intercellular spaces and disordered morphology were detected. And betaine treatment could alleviate the neuronal damage induced by HMD, restored cell morphology, and increased the number of neurons (Figure 2B). In summary, betaine attenuated HMD-induced hippocampal pathology and exerted a significant neuroprotective effect.

To determine whether betaine exerts its neuroprotective effects by reducing Hcy concentrations, we measured Hcy levels in the blood and brain of mice using ELISA. As it showed in Figure 2C, compared with the control group, betaine treatment significantly reduced the elevated concentrations of Hcy observed in the brains and blood of mice in the HMD group (Figure 2C). Additionally, we also evaluated potential organ toxicity by calculating organ indices for the brain, heart, liver, and kidneys. The results showed no significant differences in the indices of these organs with or without betaine treatment (Figure 2D). Collectively, all these findings suggested that neither HMD feeding nor betaine administration induced overt toxicity in the major organs examined during the experimental period.

### 3.3. Betaine Ameliorates HMD-Induced Hippocampal Oxidative Damage

**Figure 3:**
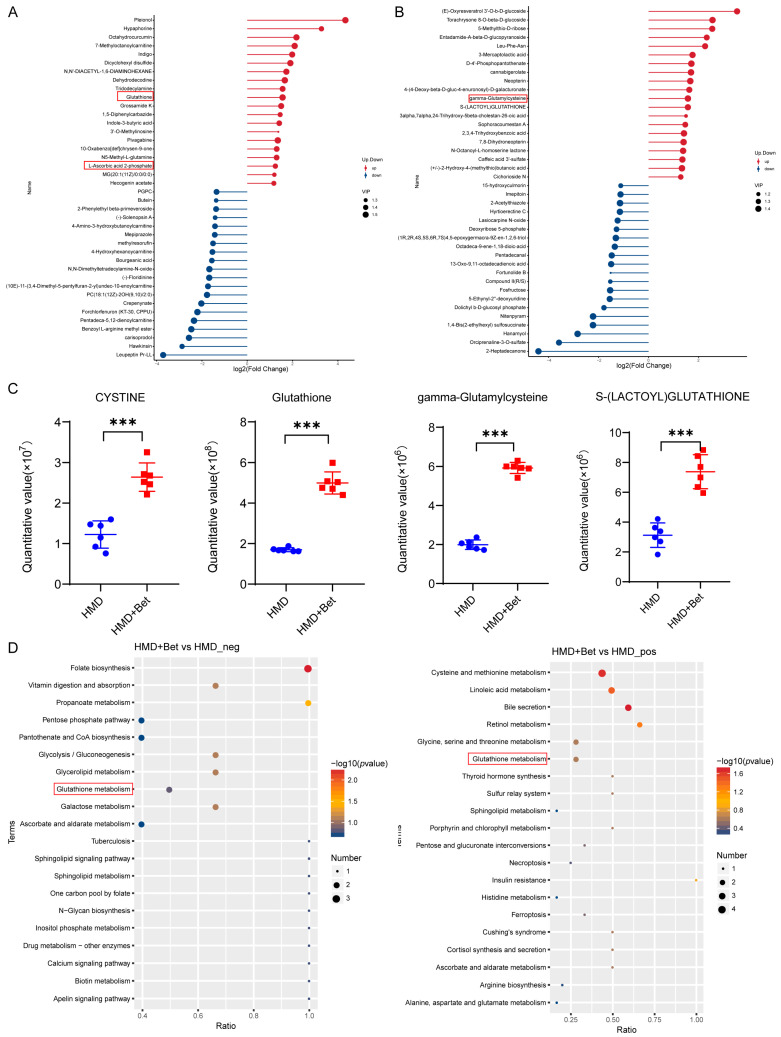
Betaine improves oxidative damage in mouse hippocampus induced by high methionine diet. (**A**,**B**) Matchstick diagram illustrating differential metabolites in positive ion (**left** panel) and negative ion (**right** panel) modes between high methionine diet mice with or without betaine rescue. The horizontal axis of stem represents the log_2_FC value, the vertical axis represents the metabolite, and the size of the points in the figure represents the VIP value. Red indicates significant upregulation, and blue indicates significant downregulation. VIP denotes variable importance in projection. The red boxes represent substances related to oxidative stress. (**C**) Quantification of ROS-related metabolites in the high methionine diet mice with or without betaine rescue. (**D**) Differentially abundant metabolite KEGG pathway enrichment analysis was used to identify the top 20 differential pathways between the high methionine diet mice with or without betaine rescue in positive and negative ion modes. The red box indicates glutathione metabolism. *** *p* < 0.001 (iIndependent- sample *t* test).

Earlier research has demonstrated that increased Hcy levels are linked to cognitive impairment [43]. To investigate the impact of betaine on the hippocampal metabolome, we performed LC–MS analysis. Among the top 20 differentially expressed metabolites identified across positive and negative ionization modes, the levels of two potent antioxidants—glutathione and L-ascorbic acid 2-phosphate—were significantly increased in the positive ion mode following betaine supplementation (Figure 3A), suggesting an increase in antioxidant defense mechanisms. Notably, in the negative ion mode, the levels of γ-glutamylcysteine, the immediate precursor of glutathione, also increased after betaine treatment (Figure 3B), thus supporting the role of betaine in mitigating HMD-induced oxidative stress. We subsequently quantified cystine, glutathione, and γ-glutamylcysteine after betaine intervention. Compared with the HMD group, all three compounds were significantly elevated following betaine treatment (Figure 3C), indicating that betaine restored glutathione synthesis and storage capacity, reinforcing its antioxidant role against Hcy-induced damage. Furthermore, S-(lactoyl) glutathione, which plays an important role in cellular protection against oxidative stress, also increased significantly (Figure 3C). These findings indicated that oxidative stress induced by hHcy could be counteracted by betaine supplementation.

Furthermore, to gain insights into the metabolic pathways underlying cognitive impairment, we conducted a KEGG pathway enrichment analysis of the differentially expressed metabolites. In both the betaine-supplemented group and the HMD group, the glutathione metabolism pathway was significantly enriched in both ionization modes (Figure 3D). These results suggested that HMD might cause cognitive impairment through oxidative stress and that betaine supplementation could ameliorate cognitive impairment by enhancing antioxidant defense.

### 3.4. Association of Betaine with PI3K/Akt/GSK-3β Pathway Modulation and Reduced Neuronal Apoptosis and Tau Hyperphosphorylation in HMD Mice

**Figure 4:**
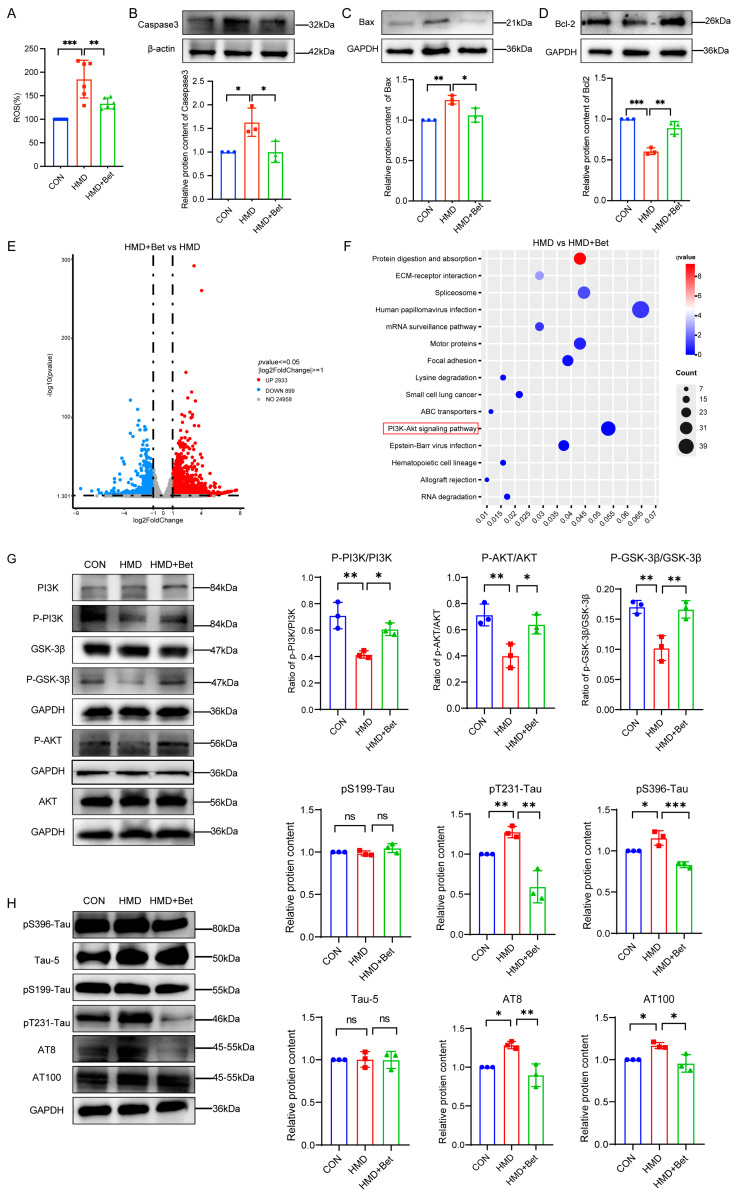
Betaine modulates the PI3K/Akt/GSK-3β signaling pathway to ameliorate neuronal apoptosis and reduce Tau protein hyperphosphorylation. (**A**) The relative ratio of ROS content in the brains of control, high-methionine diet mice with or without betaine rescue (*n* = 6). (**B**–**D**) Caspase3, Bax and Bcl-2 protein levels in the hippocampus were measured via Western blotting in control mice, high-methionine diet mice with or without betaine rescue (**upper** panel). Relative protein expression of Caspase3, Bax and Bcl-2 in the hippocampus of control, high-methionine diet mice with or without betaine rescue (**down** panel). (**E**) Volcano plot of differentially expressed genes in the hippocampus of mice fed a betaine-supplemented diet compared to those on an HMD diet. Red and blue denote significantly upregulated and downregulated genes, respectively. (**F**) KEGG pathway enrichment analysis of differentially expressed genes in hippocampal tissues of high-methionine diet-fed mice and betaine-rescued mice using RNA-seq. Red box highlights the PI3K/AKT pathway, identified as the significantly enriched target. (**G**) Western blotting was used to analyze the expression levels of p-PI3K/PI3K, p-AKT/ AKT and p-GSK-3β/GSK-3β in control mice, high-methionine diet mice with or without betaine rescue. (**H**) S199-Tau, T231-Tau, S396-Tau, Tau-5, AT8, AT100 protein levels in the hippocampus of control mice, high-methionine diet mice with or without betaine rescue were measured via Western blotting (**left** panel). Relative protein expression of S199-Tau, T231-Tau, S396-Tau, Tau-5 in the hippocampus of control mice, high-methionine diet mice with or without betaine rescue (**right** panel). The results are displayed as mean ± SD, *n* = 3. * *p* < 0.05, ** *p* < 0.01, *** *p* < 0.001; ns, not significant, (one-way ANOVA).

Increasing evidence indicates that hHcy can lead to oxidative stress, which results in apoptosis and impaired cognition [44,45,46]. To verify whether betaine could reduce oxidative stress and apoptosis in HMD-fed mice, we detected the concentration of ROS in the mouse brain. It showed ROS levels were significantly elevated in the brains of HMD-treated mice, whereas betaine treatment reduced them (Figure 4A). Next, we assessed the expression of the pro-apoptotic protein Bax and the anti-apoptotic proteins Bcl-2 and caspase-3 in the hippocampus by Western blotting. The results revealed in HMD-treated mice the Bcl-2 level was significantly decreased, whereas caspase-3 and Bax expression were increased. Betaine treatment reversed these changes (Figure 4B–D). These results suggested that betaine could inhibit apoptosis induced by oxidative stress in the hippocampus of HMD-fed mice.

To elucidate the mechanisms underlying betaine-mediated amelioration of HMD-induced cognitive impairment, we performed transcriptomic sequencing of mouse hippocampal tissue. Compared with the HMD group, betaine treatment identified 3832 differentially expressed genes, of which 2933 were upregulated and 899 downregulated (Figure 4E). According to KEGG pathway enrichment, the PI3K-Akt signaling pathway was among the top 15 significantly enriched pathways following betaine intervention, suggesting its involvement in the neuroprotective effects of betaine (Figure 4F).

It has been reported PI3K/Akt/GSK-3β signaling pathway played crucial roles in regulating the cell cycle, proliferation, apoptosis, and autophagy [24]. To investigate the role of the PI3K/Akt/GSK-3β pathway in the apoptosis of mice with cognitive impairment, we detected the phosphorylation of PI3K, Akt, and GSK-3β to assess its activation by Western blotting. It showed in HMD-fed mice, phosphorylation of both PI3K and Akt were decreased in the brain compared with controls, and betaine co-treatment reversed hyperhomocysteinemia-induced these changes whereas betaine alone did not show any significant improvements (Figure 4G and Figure S2). The PI3K/Akt pathway inhibits GSK-3β activity by phosphorylating it at Ser9. Consistent with this, betaine treatment significantly increased GSK-3β Ser9 phosphorylation (Figure 4G). To determine whether betaine reduces Tau hyperphosphorylation via the PI3K/Akt/GSK-3β pathway, we assessed Tau phosphorylation at multiple sites by Western blotting. In HMD-fed mice, phosphorylation at Ser396, Thr231, AT8 and AT100 was elevated compared with controls, indicating increased phosphorylated Tau in the brains of cognitively impaired mice. Betaine treatment significantly reduced phosphorylation at these sites in the hippocampus, whereas total Tau levels and Ser199 phosphorylation remained unchanged (Figure 4H). These results demonstrate that betaine treatment is associated with reduced tau hyperphosphorylation and activation of the PI3K/Akt/GSK-3β pathway in the mouse hippocampus.

### 3.5. Betaine Ameliorates hHcy-Induced Cytotoxicity in HT-22 Cells

**Figure 5:**
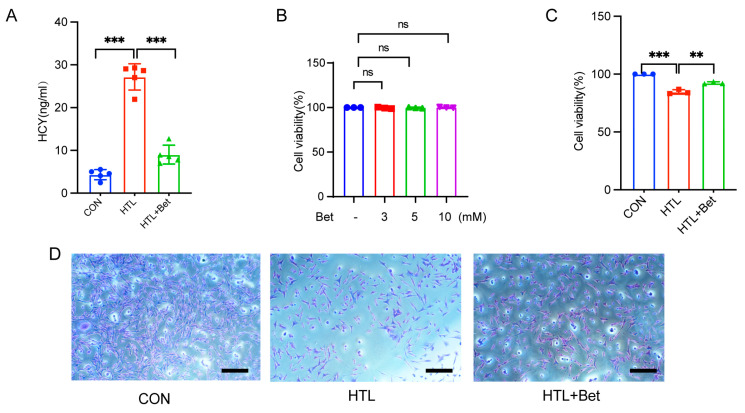
Betaine ameliorates the cytotoxicity to hHcy-induced HT-22 cells. (**A**) Hcy levels in the HT-22 cells of treated with 2 mM HTL and betaine for 24 h were measured via ELISA. (**B**) CCK-8 assay results of HT-22 cells treated with various concentrations of betaine. (**C**,**D**) Representative images of crystal violet staining and CCK-8 results after 2 mM HTL or betaine treatment in HT-22 cells. Scale bar: 200 μm. ** *p* < 0.01, *** *p* < 0.001; ns, not significant, (one-way ANOVA).

To investigate the mechanism in HMD mice in vitro, we used homocysteine thioacetamide (HTL), an intermediate metabolite in the Hcy metabolic pathway, to construct an hHcy model. First, the HT-22 cells were treated with 2 mM HTL for 24 h. ELISA revealed that compared with control cells, HT-22 cells treated with HTL presented increased Hcy levels, but after treatment with 10 mM betaine for 4 h, the Hcy content in the HT-22 cells significantly decreased (Figure 5A). To investigate the toxic effect of hHcy and betaine on HT-22 cells, we evaluated cell viability using CCK-8 and crystal violet assays. The CCK-8 results demonstrated that treatment with various concentrations of betaine did not significantly affect the viability of HT-22 cells (Figure 5B). Crystal violet assays revealed that cell viability decreased after treatment with HTL. However, betaine treatment increased the cell viability (Figure 5D). The same result was also observed for cell viability using a CCK-8 assay (Figure 5C). Collectively, these results indicated that HTL treatment led to an increase in intracellular Hcy levels, thereby decreasing cell proliferation. Interestingly, betaine treatment could reduce the intracellular Hcy concentration, thereby reducing its toxic effect on HT-22 cells.

### 3.6. Betaine Attenuates hHcy-Induced Apoptosis in HT-22 Cells by Reducing Oxidative Stress

**Figure 6:**
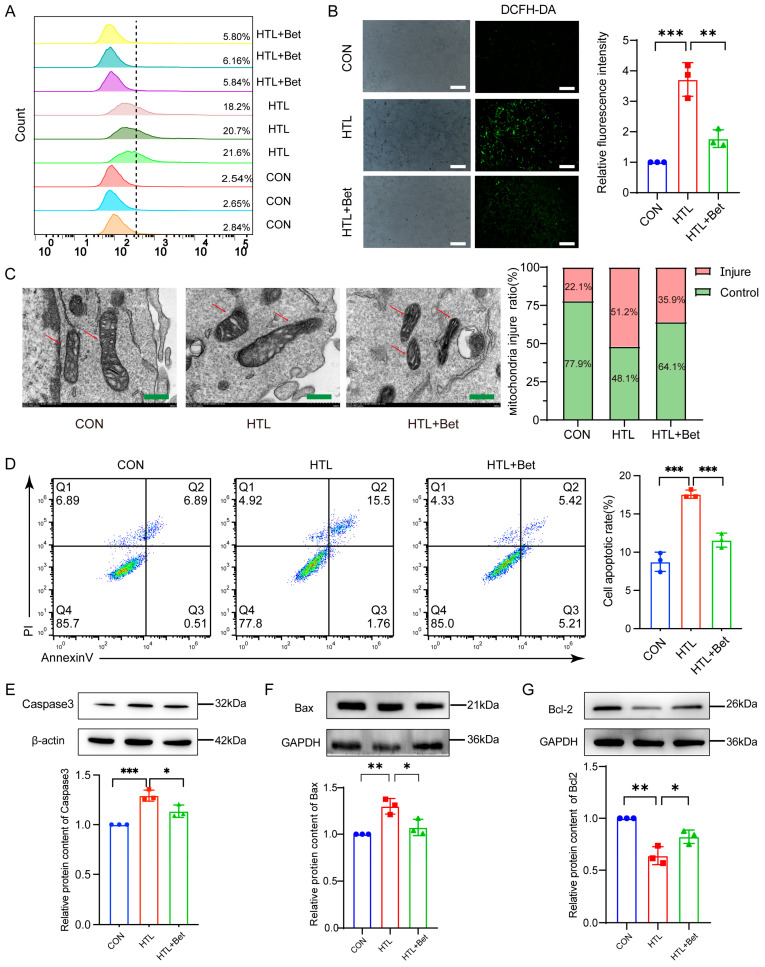
Betaine attenuates high Hcy-induced apoptosis by reducing oxidative stress in HT-22 cells. (**A**) Analysis of the changes in intracellular ROS levels in control, HTL treated HT-22 with or without betaine after 2 mM HTL for 24 h in a flow cytometry assay. CON denotes control group. HMD denotes HTL treated HT-22 and BET + HMD denotes HTL treated HT-22 cells with or without betaine. (**B**) DCFH fluorescence in control and HTL treated HT-22 cells with or without betaine. The concentration of HTL is 2 mM and for betaine is 10 mM. Scale bar: 200 μm. DCFH denotes 2′,7′-dichlorofluorescin. (**C**) Mitochondrial morphology of HT-22 cells after HTL and betaine treatment, shown by representative TEM images. The mitochondrial damage scoring criteria were referenced from [48]. Scale bar: 500 μm. The red arrows indicate representative mitochondria. (**D**) The apoptosis of control and HTL treated HT-22 cells with or without betaine by flow cytometry. In the dot plots, blue indicates viable cells (Annexin V^−^/PI^−^), green indicates early apoptotic cells (Annexin V^+^/PI^−^), and red indicates late apoptotic/necrotic cells (Annexin V^+^/PI^+^). (**E**–**G**) Caspase3, Bax and Bcl-2 protein levels in the control and HTL treated HT-22 cells with or without betaine were measured via Western blotting (**upper** panel). Relative protein expression of caspase3, Bax and Bcl-2 in the control and HTL treated HT-22 cells with or without betaine (**down** panel). The results are displayed as mean ± SD, *n* = 3. * *p* < 0.05, ** *p* < 0.01, *** *p* < 0.001. (one-way ANOVA).

hHcy has been reported to lead to an increase in cellular oxidative stress, which in turn causes changes in apoptosis [47]. To investigate whether betaine affects oxidative stress, we measured intracellular ROS levels in HT-22 cells treated with HTL. Flow cytometry and immunofluorescence analyses showed that HTL significantly increased ROS levels, an effect reversed by betaine treatment (Figure 6A,B). Consistent with the role of mitochondria as primary targets of ROS-induced damage, we next examined whether betaine preserves mitochondrial ultrastructure. Transmission electron microscopy (TEM) revealed that mitochondria in control HT-22 cells exhibited typical elongated or oval shapes with well-defined cristae and a homogeneous matrix. In contrast, HTL exposure induced marked mitochondrial abnormalities, including swelling, spherical transformation, cristae fragmentation or loss, matrix vacuolization, and outer membrane disruption. Betaine treatment largely prevented these alterations (Figure 6C). These findings suggested that betaine not only mitigated HTL-induced ROS bursts but also preserved mitochondrial ultrastructural integrity.

Next, we evaluated the apoptosis of the HTL-treated HT-22 cells with an Annexin V/PI assay. The results showed that HTL caused apoptosis in HT-22 cells. After treatment with betaine, apoptosis significantly decreased (Figure 6D). We next assessed the apoptosis marker caspase-3 by Western blotting. HTL treatment increased caspase-3 levels, whereas betaine reversed this effect. (Figure 6E). We further assessed the Bcl-2/Bax ratio by western blotting. HTL treatment decreased the Bcl-2 protein levels and increased the Bax levels, while these effects were reversed by betaine treatment (Figure 6F,G). All these results suggested that HTL induced HT-22 apoptosis via ROS and that betaine rescued this apoptosis.

### 3.7. Betaine Attenuates Tau Aggregation Through PI3K/AKT/GSK-3β Pathway

**Figure 7:**
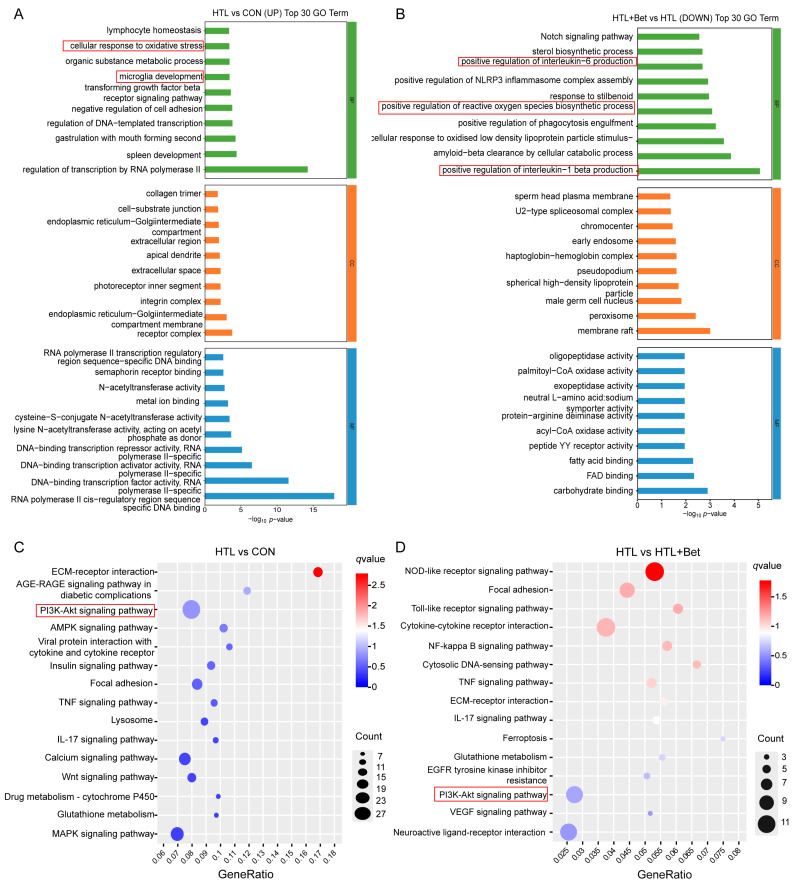
GO and KEGG enrichment analyses of differentially expressed genes in HT-22 cells treated with betaine and HTL. (**A**,**B**) GO analysis of upregulated genes in HTL-treated cells compared with controls (**left** panel) and downregulated genes in betaine-treated cells compared with HTL-treated cells (**right** panel) based on RNA-seq data. BP represents a biological process term in the GO analysis. CC represents a cellular component term in the GO analysis. MF represents a molecular function term in the GO analysis. B-HTL denotes HTL treated HT-22 with betaine rescue. Red boxes highlight GO analysis of interest. (**C**,**D**) KEGG analysis of differentially expressed genes between HTL-treated and control cells, as well as between betaine-treated and HTL-treated cells. The red box indicates the PI3K-Akt signaling pathway.

To elucidate the mechanism by which betaine protects against cognitive impairment, we performed RNA-seq on HTL-treated HT-22 cells with or without betaine rescue. The PI3K/Akt pathway is known to regulate GSK-3β-mediated Tau hyperphosphorylation and apoptosis in AD [49]. RNA-seq analysis revealed that genes upregulated by HTL treatment were enriched in pathways related to microglial development and oxidative stress (Figure 7A). Conversely, genes downregulated by betaine treatment were associated with positive regulation of interleukin-6 production, interleukin-1β production, and reactive oxygen species biosynthesis (Figure 7B). KEGG pathway analysis revealed the PI3K-Akt signaling pathway ranked within the top 15 most enriched pathways in HTL-treated HT-22 cells (Figure 7C). This pathway was similarly enriched following betaine treatment (Figure 7D). Together, these results confirm that the PI3K-Akt pathway is involved in hHcy-induced cognitive impairment and suggest that betaine exerts its protective effects through this pathway.

**Figure 8:**
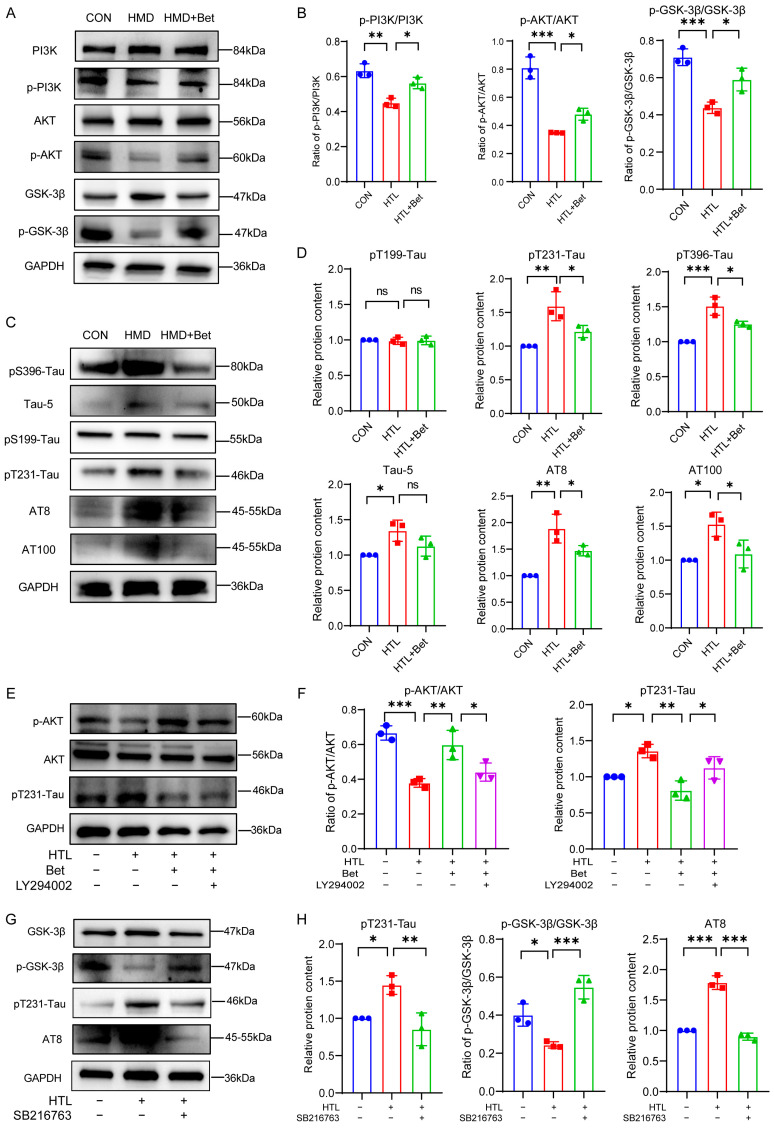
Betaine attenuates tau protein aggregation through the PI3K/AKT/GSK-3β pathway. (**A**,**B**) The expression levels of p-PI3K/PI3K, p-AKT/AKT and p-GSK-3β/GSK-3β in control and HTL treated HT-22 cells with or without betaine measured by Western blot (**left** panel). Statistical plots of the expression levels of p-PI3K/PI3K, p-AKT/AKT and p-GSK-3β/GSK-3β by Western blotting (**right** panel). (**C**,**D**) S199-Tau, T231-Tau, S396-Tau, Tau-5, AT8, AT100 protein levels in the control and HTL treated HT-22 cells with or without betaine (**left** panel). Statistical plots of the expression levels of S199-Tau, T231-Tau, S396-Tau and Tau-5 by Western blotting (**right** panel). (**E**,**F**) p-AKT/AKT and T231-Tau protein expression levels in the presence of LY294002 or not (**left** panel). Statistical plots of the expression levels of p-AKT/AKT and T231-Tau by Western blotting (**right** panel). (**G**,**H**) p-GSK-3β/GSK-3β, AT8 and T231-Tau protein expression levels in the presence of SB216763 or not (**left** panel). Statistical plots of the expression levels of p-GSK-3β/GSK-3β, AT8 and T231-Tau by Western blotting (**right** panel). The results are displayed as mean ± SD, *n* = 3. * *p* < 0.05, ** *p* < 0.01, *** *p* < 0.001; ns, not significant, (one-way ANOVA).

The PI3K/AKT/GSK-3β pathway plays a pivotal role in neuroprotection by promoting cell survival and inhibiting apoptosis [50]. To investigate whether betaine modulates Tau hyperphosphorylation via this pathway in HT-22 cells, we assessed the phosphorylation levels of PI3K, Akt, and GSK-3β by Western blotting. HTL treatment significantly reduced the p-PI3K/PI3K, p-Akt/Akt, and p-GSK-3β/GSK-3β ratios, whereas betaine treatment restored these levels, consistent with observations in HMD-fed mice (Figure 8A,B). In parallel, HTL treatment increased Tau phosphorylation at Ser396, Thr231, AT8, AT100, an effect reversed by betaine without altering total Tau levels (Figure 8C,D). Then, to determine whether the PI3K/AKT pathway is required for the neuroprotective effects of betaine, we pretreated HT-22 cells with LY294002 (20 μmol/L) 1 h prior to betaine administration. Western blot analysis revealed that LY294002 co-treatment, not only abrogated betaine-induced AKT phosphorylation but also reversed its inhibitory effect on Tau phosphorylation at Thr231, restoring p-Tau levels to those observed in the HTL group (Figure 8E,F and Figure S3). To further validate the critical role of GSK-3β in HTL-induced tau phosphorylation and misfolding, the selective GSK-3β inhibitor SB216763 was used. HT-22 cells were treated with HTL for 24 h with or without SB216763. Western blot analysis (Figure 8G,H) showed that, compared with the HTL group, SB216763 treatment significantly restored p-GSK-3β (Ser9) levels and decreased tau phosphorylation at both Thr231 and the AT8 epitope (Ser202/Thr205). These results demonstrate that pharmacological inhibition of GSK-3β effectively blocks HTL-induced tau phosphorylation, supporting that GSK-3β as a critical mediator in hyperhomocysteinemia-related tau pathology. Together, these results indicate that PI3K/AKT/ GSK-3β activity is essential for betaine-mediated suppression of Tau hyperphosphorylation, positioning this pathway as a central mediator of betaine’s protective effects against hHcy-induced pathology.

### 3.8. ROS Modulates the PI3K/AKT Pathway in HTL-Induced Apoptosis

**Figure 9:**
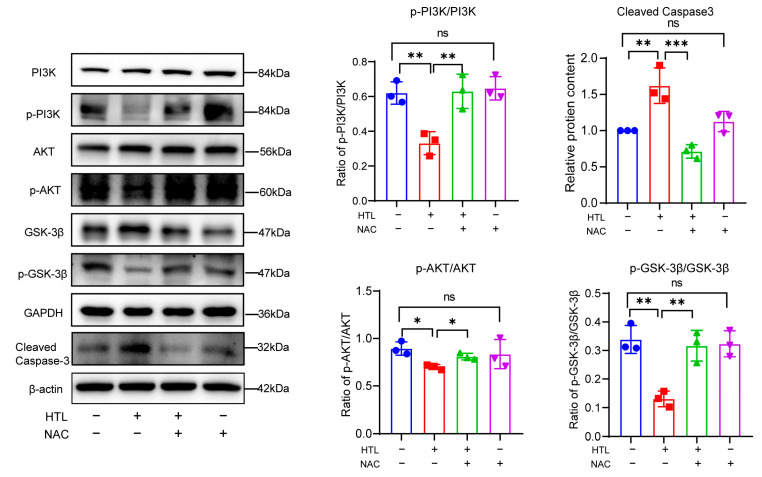
Modulation of the PI3K/AKT signaling pathway by ROS in HTL-induced apoptosis. Western blotting was performed to detect the expression levels of p-PI3K/PI3K, p-AKT/AKT, p-GSK-3β/GSK-3β and Cleaved Caspase-3 (**left** panel), with corresponding statistical plots (**right** panel). The results are displayed as mean ± SD, *n* = 3. * *p* < 0.05, ** *p* < 0.01, *** *p* < 0.001; ns, not significant, (one-way ANOVA).

To further confirm the relationship between the levels of ROS and the activity of the PI3K/AKT signaling pathway, the expression levels of the corresponding proteins were measured. Notably, co-treatment with the ROS scavenger NAC significantly reversed the HTL-induced inhibition of this pathway, as evidenced by elevated p-PI3K/PI3K, p-AKT/AKT, and p-GSK-3β/GSK-3β ratios, along with a marked reduction in cleaved caspase-3 levels. In summary, these results suggested that HTL triggered apoptotic cell death by increasing ROS levels and blocking the activity of the PI3K/AKT/GSK-3β signaling pathway, a pathogenic process that was markedly alleviated by NAC (Figure 9A). Collectively, these in vitro results indicate that hHcy promotes neurotoxicity, an effect alleviated by NAC.

## 4. Discussion

**Figure 10:**
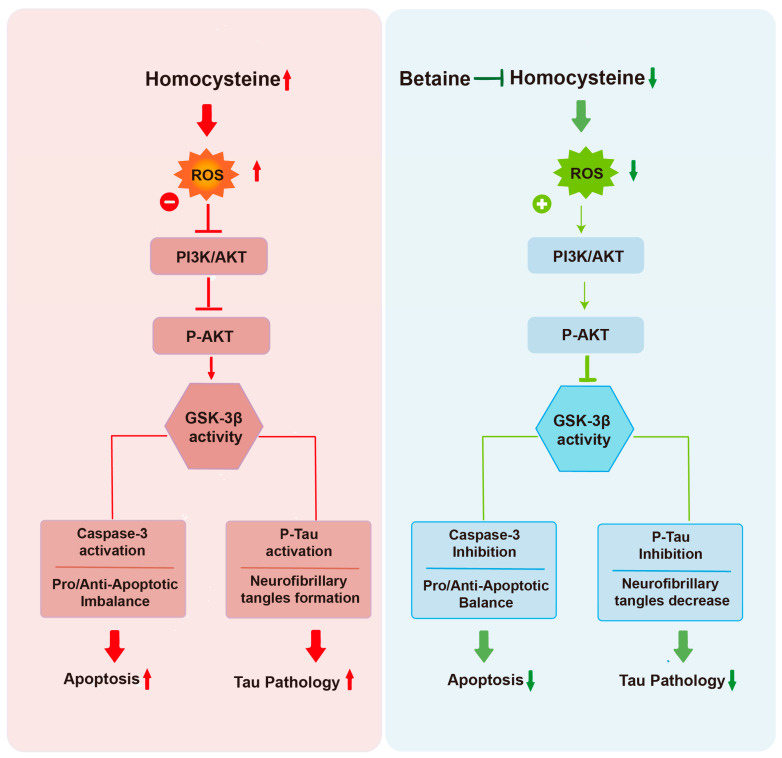
Schematic diagram of the mechanism by which betaine alleviates ROS-mediated PI3K/AKT/GSK-3β signaling. Arrows indicate the direction of regulation; ‘+’ indicates activation, ‘−’ indicates inhibition.

Hcy is a widely recognized risk factor for cognitive decline [42] and reducing Hcy levels offers a promising strategy for preventing dementia. Although betaine is known to reduce Hcy levels, its protective effects against Hcy-induced cognitive impairment and the underlying mechanisms remain largely unexplored. Here, we show that betaine attenuates Hcy-induced cognitive impairment by reducing ROS levels and suppressing oxidative stress. Mechanistically, betaine modulates the PI3K/AKT/GSK-3β pathway to ameliorate neuronal apoptosis and reduce Tau hyperphosphorylation, both in vivo and in vitro (Figure 10). These findings suggest that betaine supplementation may serve as an effective intervention for alleviating Hcy-associated cognitive impairment.

Folic acid is widely used to treat hyperhomocysteinemia. However, its clinical efficacy in improving hHcy-related cognitive impairment remains controversial [51,52]. This limited efficacy is largely attributable to the poor brain bioavailability of folate. To exert its effects in the central nervous system, folate must cross the blood–cerebrospinal fluid barrier or the blood–brain barrier; however, its transport efficiency varies considerably among individuals [53]. Furthermore, genetic polymorphisms in folate transporters, such as RFC/SLC19A1, can further disrupt cerebral folate metabolism and transport, resulting in substantial interindividual variability in brain folate levels even under standardized supplementation regimens. These factors severely constrain the universal therapeutic value of folate for hHcy-associated cognitive disorders [54]. By contrast, betaine acts through the BHMT pathway, which operates independently of folate metabolism to directly lower hcy levels, thereby providing an alternative intervention that overcomes the issue of poor folate availability in the brain [55].

The main physiological action of betaine is to serve as a methyl donor (transmethylation), which leads to an increase in SAM levels [56]. These findings indicate that betaine supplementation can effectively decrease plasma Hcy levels [57], which is consistent with our findings in HMD-fed mice and in HT-22 cells treated with HTL. Betaine also exerts protective effects in the brain and other organs through antioxidant and anti-inflammatory pathways. In patients with AD, betaine treatment improved cognition and function after one month, accompanied by reduced blood levels of Hcy and the inflammatory cytokines IL-1β and TNF-α [58]. In mice fed HMD for two weeks, betaine reduced hippocampal ROS levels and increased glutathione, cystine, and γ-glutamylcysteine. Consistently, GO analysis of the hippocampal metabolome revealed enrichment of glutathione metabolism. Together, these results indicate that betaine effectively restores glutathione biosynthesis, which is impaired under Hcy-induced oxidative stress. This restoration is supported by increased levels of the precursor amino acid cystine, enhanced catalytic capacity of the rate-limiting step reflected by elevated γ-glutamylcysteine, and consequent replenishment of the cellular glutathione pool. These findings provide direct molecular evidence that betaine alleviates Hcy-induced oxidative stress and cognitive impairment by enhancing endogenous antioxidant defense.

Recent evidence has identified hHcy as a risk factor for neurodegenerative disease. Consistently, our previous study showed that HMD-induced hHcy in mice leads to nerve injury, inflammation, and Tau aggregation [42]. To investigate whether betaine alleviates Hcy-induced oxidative stress and cognitive impairment, we evaluated apoptosis and Tau pathology in vivo and in vitro. Both approaches showed that betaine attenuated hHcy-induced apoptosis, as evidenced by reversal of increased caspase-3 and Bax levels and restoration of decreased Bcl-2 levels in the brain and HT-22 cells. Additionally, Western blotting revealed that betaine reduced phosphorylation of Tau at Thr231 and Ser396—markers associated with cognitive dysfunction—which were elevated in the hHcy brain. The PI3K/Akt/GSK-3β pathway plays a vital neuroprotective role in AD [59] and aberrant GSK-3β activation is known to promote Tau hyperphosphorylation, apoptosis, and inflammation [60]. Our analysis showed that the PI3K/AKT signaling pathway was enriched in hHcy-treated HT-22 cells regardless of betaine rescue. We therefore investigated whether betaine rescues hHcy-induced cognitive impairment by modulating this pathway. In vivo and in vitro results consistently demonstrated that hHcy increased phosphorylation of PI3K, Akt, and GSK-3β, whereas betaine reversed these effects.

Together, these data indicate that betaine improves cognitive function by reducing apoptosis and Tau pathology through modulation of the PI3K/AKT/GSK-3β signaling pathway. This study has several limitations. First, the lack of human brain samples restricts clinical validation. Second, no genetic or pharmacological manipulation of the PI3K/Akt/GSK-3β pathway was performed in the mouse model. Therefore, future studies using brain-specific conditional knockout mice or intracranial delivery of pathway inhibitors are required to provide direct evidence for the necessity of this pathway in vivo. Recent studies have identified additional targets for betaine, including direct inhibition of TBK1 [36] and suppression of NLRP3-mediated pyroptosis via m6A-YTHDF2 [37]. While these pathways are distinct from the ROS/PI3K/AKT/GSK-3β/tau axis we report here, they collectively suggest that betaine may exert pleiotropic neuroprotective effects through multiple parallel mechanisms depending on the pathological context. In conclusion, our study demonstrates that betaine reduces ROS levels and enhances endogenous antioxidant defense, thereby inhibiting apoptosis and Tau aggregation through modulation of the PI3K/Akt/GSK-3β pathway, ultimately alleviating Hcy-induced cognitive impairment. Betaine supplementation may therefore represent a candidate approach that warrants further investigation for Hcy-related cognitive impairment. Together, these findings provide preclinical evidence supporting the potential of betaine for improving cognitive function, although long-term efficacy, dose–response relationships, and comprehensive safety assessments remain to be established.

## Data Availability

The RNA-seq data for this article are available in the GEO database under accession number GSE308639 at https://www.ncbi.nlm.nih.gov/geo/query/acc.cgi?acc=GSE308639, accessed on 25 June 2026.

## Supplementary Materials

The following supporting information can be downloaded at: https://www.mdpi.com/article/10.3390/antiox15070807/s1, Figure S1: Betaine alone does not affect cognitive function. A Heat maps of the T maze test in control and high methionine diet group with or without betaine treatment. B, C The time and the number of new arms entered by the control or high methionine diet groups with or without betaine rescue mice in the T maze test (*n* = 6). D Group average heatmap of the swimming trajectories of the control or betaine mice in Morris water maze tests (*n* = 6). E–G Escape latency during the training period, latency to first platform crossing and number of platform location crosses during the Morris water maze experimental period (*n* = 6). CON denotes control group. Betaine denotes mice received betaine alone. * *p* < 0.05, ** *p* < 0.01, *** *p* < 0.001. Figure S2: Betaine alone has no significant effect on PI3K/AKT pathway activity or tau phosphorylation. A, B Representative Western blots (left) and quantification (right) of p-AKT/AKT, AT8, and T231-Tau in mouse hippocampus treated with betaine alone compared with normal control. C, D Representative Western blots (left) and quantification (right) of p-AKT/AKT, AT8, and T231-Tau in HT-22 cells treated with betaine alone compared with normal control. The results are displayed as mean ± SD, *n* = 3. * *p* < 0.05, ** *p* < 0.01, *** *p* < 0.001. Figure S3: Effect of LY294002 alone on PI3K/AKT pathway and tau phosphorylation. A, B Representative Western blots (left) and quantification (right) of p-AKT/AKT and T231-Tau in HT-22 cells treated with LY294002 alone compared with normal control. The results are displayed as mean ± SD, *n* = 3. * *p* < 0.05, ** *p* < 0.01, *** *p* < 0.001.
