# Supplementary material for: Betaine Attenuates Hyperhomocysteinemia-Induced Cognitive Impairment by Suppressing Oxidative Stress and Activating the PI3K/AKT/GSK-3β Pathway
Source: Antioxidants (Basel). 2026 Jun 27;15(7):807. doi: 10.3390/antiox15070807 (PMC13405995; doi:10.3390/antiox15070807)
Supplement: Supplementary file 1 [file antioxidants-15-00807-s001.zip › antioxidants-4259543-supplementary.pdf]

Supplementary Materials

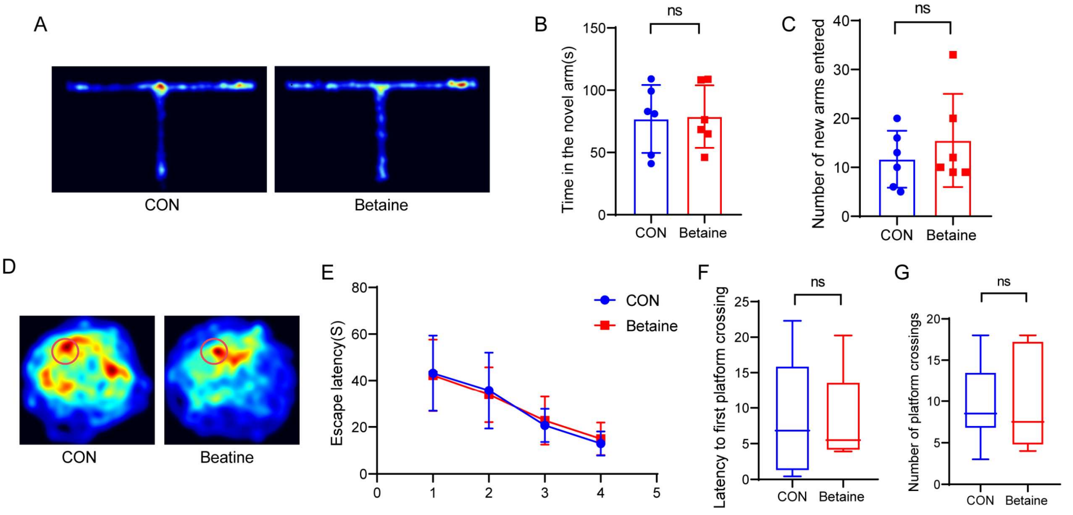

FigureS1 Betaine alone does not affect cognitive function

A Heat maps of the T maze test in control and high methionine diet group with or without betaine treatment. **B-C** The time and the number of new arms entered by the control or high methionine diet groups with or without betaine rescue mice in the T maze test (*n* = 6). **D** Group average heatmap of the swimming trajectories of the control or betaine mice in morris water maze tests (*n* = 6). **E-G** Escape latency during the training period, latency to first platform crossing and number of platform location crosses during the morris water maze experimental period (*n* = 6). CON denotes control group. Betaine denotes mice received betaine alone. *P<0.05, **P<0.01, ***P<0.001.

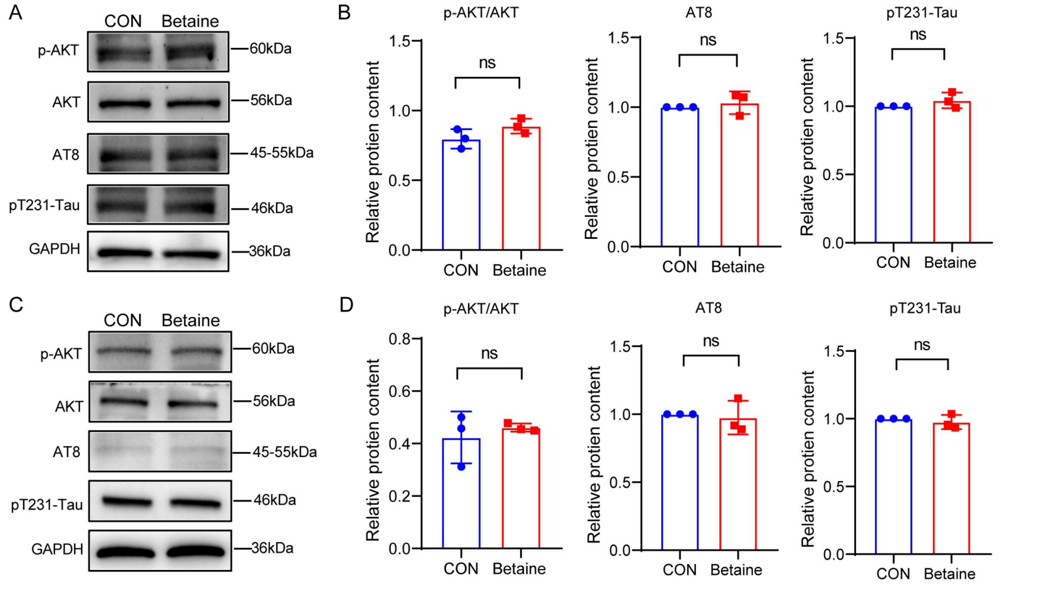

Figure S2 Betaine alone has no significant effect on PI3K/AKT pathway activity or tau phosphorylation.

A-B Representative Western blots (left) and quantification (right) of p-AKT/AKT, AT8, and T231-Tau in mouse hippocampus treated with betaine alone compared with normal control. **C-D** Representative Western blots (left) and quantification (right) of p-AKT/AKT, AT8, and T231-Tau in HT-22 cells treated with betaine alone compared with normal control. The results are displayed as mean ± SD, *n* = 3. *P < 0.05, **P < 0.01, *** P < 0.001.

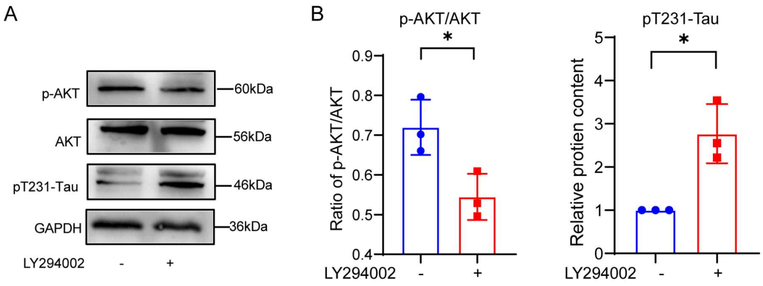

Figure S3 Effect of LY294002 alone on PI3K/AKT pathway and tau phosphorylation.

A-B Representative Western blots (left) and quantification (right) of p-AKT/AKT and T231-Tau in HT-22 cells treated with LY294002 alone compared with normal control. The results are displayed as mean ± SD, $n = 3$. *P < 0.05, **P < 0.01, *** P < 0.001.
